# Altered Spatial Organization of Dynamic Functional Network Associates With Deficient Sensory and Perceptual Network in Schizophrenia

**DOI:** 10.3389/fpsyt.2021.687580

**Published:** 2021-08-05

**Authors:** Hui He, Cheng Luo, Chuan He, Manxi He, Jing Du, Bharat B. Biswal, Dezhong Yao, Gang Yao, Mingjun Duan

**Affiliations:** ^1^The Clinical Hospital of Chengdu Brain Science Institute, Ministry of Education (MOE) Key Lab for Neuroinformation, University of Electronic Science and Technology of China, Chengdu, China; ^2^Research Unit of NeuroInformation, Chinese Academy of Medical Sciences, Chengdu, China; ^3^Department of Biomedical Engineering, New Jersey Institute of Technology, Newark, NJ, United States

**Keywords:** schizophrenia, fMRI, dynamic functional connectivity, ICA, spatial organization

## Abstract

Schizophrenia is currently thought as a disorder with dysfunctional communication within and between sensory and cognitive processes. It has been hypothesized that these deficits mediate heterogeneous and comprehensive schizophrenia symptomatology. In this study, we investigated as to how the abnormal dynamic functional architecture of sensory and cognitive networks may contribute to these symptoms in schizophrenia. We calculated a sliding-window-based dynamic functional connectivity strength (FCS) and amplitude of low-frequency fluctuation (ALFF) maps. Then, using group-independent component analysis, we characterized spatial organization of dynamic functional network (sDFN) across various time windows. The spatial architectures of FCS/ALFF-sDFN were similar with traditional resting-state functional networks and cannot be accounted by length of the sliding window. Moreover, schizophrenic subjects demonstrated reduced dynamic functional connectivity (dFC) within sensory and perceptual sDFNs, as well as decreased connectivity between these sDFNs and high-order frontal sDFNs. The severity of patients' positive and total symptoms was related to these abnormal dFCs. Our findings revealed that the sDFN during rest might form the intrinsic functional architecture and functional changes associated with psychotic symptom deficit. Our results support the hypothesis that the dynamic functional network may influence the aberrant sensory and cognitive function in schizophrenia, further highlighting that targeting perceptual deficits could extend our understanding of the pathophysiology of schizophrenia.

## Introduction

Schizophrenia is a disabling mental disorder that affects about one percentage of the world's adult population (1). Schizophrenic patients 1are normally characterized by abnormalities in distinguishing between the self and other individuals and confirming whether their thoughts and actions are independent from external influences (2). Thus, the specific positive symptoms (e.g., self-disorder) have been regarded as a hallmark characteristic of schizophrenia (3, 4). Understanding the underlying neuropathology of schizophrenia is likely to be key for the development of treatment for the schizophrenic subjects. So far, neuroimaging has provided abundant evidence for the dysconnectivity hypothesis, implying brain functional disintegration in schizophrenia (5, 6). Yet, due to frequent observations of sensory and perceptual symptoms in schizophrenic subjects, the pathophysiology of schizophrenia has been attributed to disintegrated sensory and cognitive functional processing (7–9).

Altered integration of functional connectivity in multisensory processing may contribute to mapping the neural pathophysiology of schizophrenia (7, 10). Combined with clear symptomatology, studies have indicated that positive symptoms of schizophrenia have been attributed to impaired integration of bottom-up and top-down brain networks (7, 11, 12). For example, auditory hallucination is regarded as a failure of the top-down inhibitory control of bottom-up perceptual processes in schizophrenic subjects (11). Therefore, it is crucial to profoundly understand the pathophysiology of schizophrenia through assessing the functional interaction within and between primary sensorimotor and high-order cognitive networks. Consequently, increased knowledge was required to dissect the pathophysiology of schizophrenia. Such mechanism and functioning can be commonly studied using the functional connectivity of BOLD (blood-oxygen-level-dependent) signals between brain regions with an implicit assumption of stationary connectivity during the scanning period (13). However, integrating the connectivity signals over a long time provides a single measure of the functional connectivity and may obscure important dynamic features of network behavior. Accumulating evidence has suggested that brain networks are dynamically connected (14, 15). Dynamic analysis could expand our current knowledge regarding dysconnectivity in schizophrenia (16).

It is noteworthy that the investigation of dynamic functional connectivity (dFC) is actively applied to investigate the changes of sensorimotor and cognitive functional processing in schizophrenia. Damaraju et al. have suggested that schizophrenic subjects showed aberrant transient states of resting-state functional connectivity within the sensorimotor network (17, 18). Moreover, the aberrant local dynamic functional feature of the visual network and its dFC is correlated in time and their correlations are altered in schizophrenia (19). A recent study has also revealed that schizophrenia was associated with the instable dFC between sensory and high-order functional networks (20). Du et al. found the reduced dynamic characteristics within the default model network (DMN) at rest overtime, which is associated with impaired ability in making self-other distinctions (21). The aforementioned studies have provided some of the first quantitative insights to unveil the deficient functional flexibility of sensorimotor and cognitive processing in schizophrenia.

The dFC architecture during tasks could be shaped by resting-state functional networks (RSNs) (22–24). Till date, few studies have investigated the resting-state spatial organization of dynamic functional network (sDFN) in the human brain. The architecture of dFC might provide functional insights into flexible behavioral functions (25), while the abnormal sDFN of schizophrenia remained unknown. Thus, it is necessary to track and assess the sDFN in schizophrenia. Liang et al. have indicated that the functional connectivity strength (FCS) metric is closely associated with physiological measures (26). The amplitude of low-frequency fluctuations (ALFF) has also been proven to be a reliable index of local intrinsic brain activity (27). Moreover, Leonardi and Van De Ville concluded that sliding widow-based dFC could truly measure the characteristics of the dynamic functional network (28). Therefore, sliding-window-based FCS and ALFF analysis could be considered as a stable way to characterizing the sDFN.

Based on resting-state fMRI, we sought to assess whether the change in architecture of dFC was associated with the pathophysiology of schizophrenia through the analysis of sDFN. We conducted sliding window-based dynamic FCS and ALFF maps and then tracked the sDFN of these functional maps across time windows through group-independent component analysis (ICA). On the basis of previous findings, we hypothesized that spatial patterns of sDFN were largely overlapped with RSN in the human brain. Moreover, we hypothesized that schizophrenic patients would show abnormal sensory and perceptual sDFN.

## Materials and Methods

### Subject Selection

The study participants comprised 102 patients diagnosed with schizophrenia and 128 healthy controls that had been matched in age and gender. The chronic schizophrenic subjects were recruited from the Clinical Hospital of Chengdu Brain Science Institute (CBSI). The patients were diagnosed using the Structured Clinical Interview for the DSM-IV Axis I disorders—clinical version (SCID-I-CV), and all patients were being treated with medication (e.g., antipsychotics). The psychiatric symptom of schizophrenia was assessed using the Positive and Negative Symptom Scale (PANSS). The exclusion criteria for healthy controls were the following: a history of medical, neuropsychiatric illness, and major neurological or psychiatric illness in their first-degree relatives. This study was approved by the Ethics Committee of the Clinical Hospital of CBSI in accordance with the Helsinki Declaration. Written informed consent was obtained from each subject before the scanning.

### Imaging Acquisition

Imaging was acquired on a 3T MRI scanner (GE Discovery MR750, GE Healthcare, Chicago, IL, USA). During scanning, foam padding and earplugs were used to reduce head motion and scanning noise, respectively. High-resolution T1-weighted images were acquired using a three-dimensional fast spoiled gradient echo sequence [repetition time (TR) = 6.008 ms, echo time (TE) = 1.98 ms, flip angle (FA) = 9°, matrix size = 256 × 256, field of view (FOV) = 256 × 256 mm^2^, slice thickness = 1 mm, no gap, 152 slices]. Subsequently, resting-state functional MRI data were acquired using gradient-echo echo planar imaging sequences (TR = 2,000 ms, TE = 30 ms, FA = 90°, matrix size = 64 × 64, FOV = 240 × 240 mm^2^, slice thickness/gap = 4 mm/0.4 mm, number of slices = 35), with an eight-channel phased-array head coil. All subjects underwent a 510-s resting-state scan to yield 255 volumes. The first 10 volumes (20 s) of data were discarded for magnetization equilibrium. During resting-state fMRI, all subjects were instructed to have their eyes closed and to move as little as possible without falling asleep.

### MRI Data Pre-processing

Functional imaging data were pre-processed using the NIT toolbox (29) according to a standard pipeline similar with our previous study (30) and only briefly described here. Slice time correction and head motion correction were carried out. Then, the spatial normalization (3 ^*^ 3 ^*^ 3 mm) of the functional images was performed using 3D T1-based transformation. Any subjects who had a maximum translation larger than 3 mm or 3° were excluded. Framewise displacement (FD) was also evaluated as suggested by Power et al. (31). To minimize the effect in fMRI due to drifts, detrending analysis was performed. Then, sources of nuisance signals were removed from the normalized images through linear regression (six motion parameters and their first temporal derivative, white matter signal, cerebrospinal fluid signal). The global signal was not regressed out (32). Finally, fMRI data were passed through a bandpass 0.01–0.08 Hz.

Structural images were processed using the SPM12 toolbox. The whole-brain gray matter (GM) and total intracranial volume (TIV) were assessed, respectively. For each subject, the GM volume was normalized by dividing the TIV score. Then, the subjects' normalized GM value was entered as a global variable in statistical analysis to correct for the variability of global GM volume across subjects.

### Dynamic Brain Functional Network Construction

**Figure 1:**
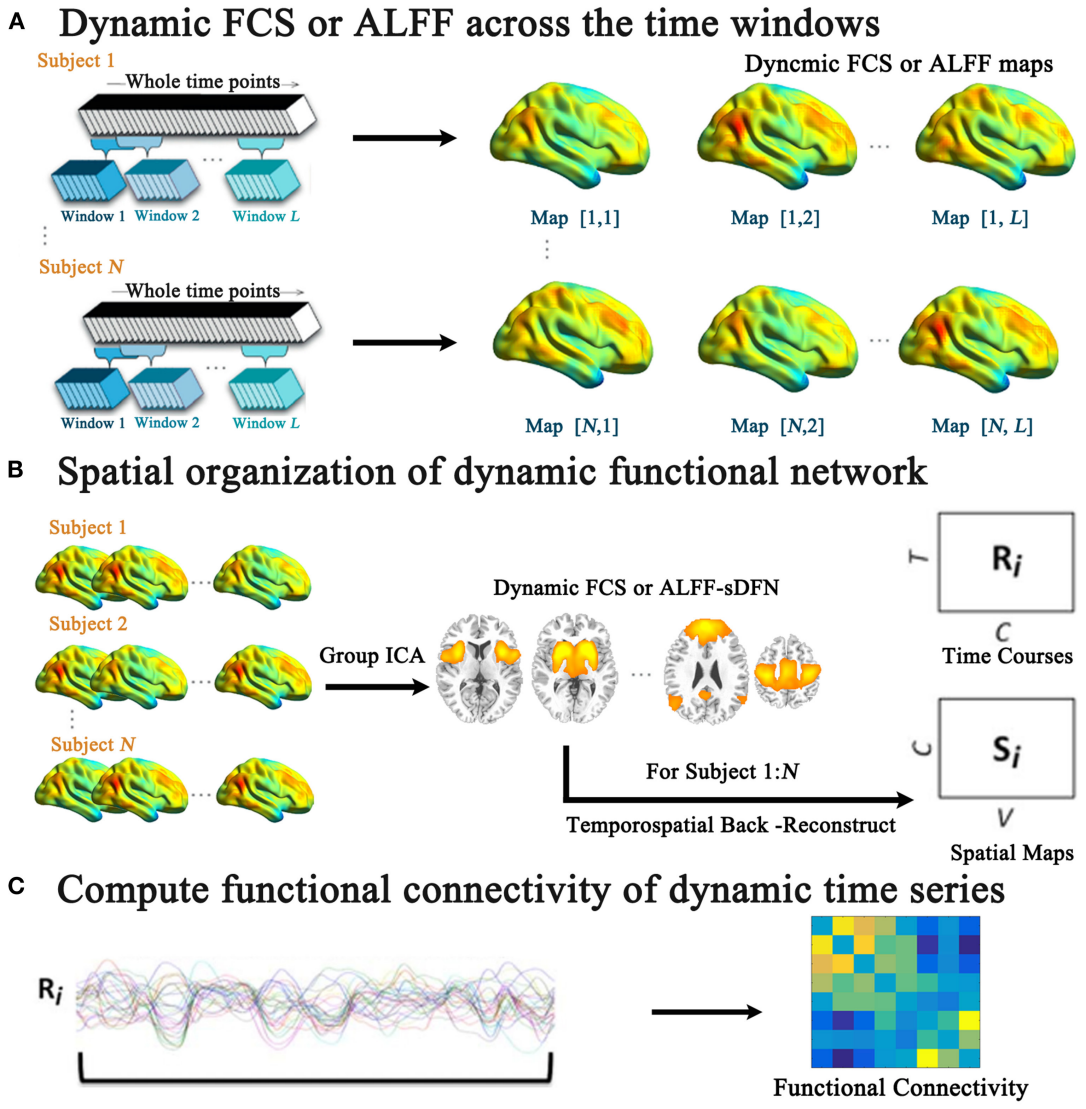
The schematic of the investigated aberrant spatial organization of dynamic functional network (sDFN) across windows in schizophrenia. **(A)** Denotes the sliding-window-based dynamic functional connectivity strength (FCS) or dynamic amplitude of low-frequency fluctuation (ALFF) analyses for each subject. Within **(B)**, the group spatial ICA was performed for dynamic FCS and ALFF maps across windows, respectively. **(C)** Denotes the functional connectivity calculation among the dynamic time series of sDFN.

For each subject, we calculated sliding window-based whole-brain dynamic functional maps across time windows, including dynamic FCS and ALFF maps (Figure 1A). First, the whole run-time series were segmented into *L* windows, sliding the onset of each window by 10 s. Due to fact that the minimum window length should be no <1/*f*_*min*_ (28), s 100-s window length was selected (*f*_*min*_ = 0.01 Hz). Then, within the *i*th time window the FCS and ALFF maps were calculated. For each subject, we can get 40 FCS and ALFF maps, respectively. The calculation steps of FCS and ALFF were as shown below.

FCS analysis was performed as follows: (1) to exclude artifactual correlations from non-GM, the GM mask was generated by thresholding (cutoff = 0.2) the average of the GM probability map involving all subjects. (2) The time course within the GM mask was extracted, and the Pearson's correlations between every pair of voxels were calculated for each subject. (3) We then transformed individual correlation matrices to a z-score matrix using a Fisher *r*-to-*z* transformation. (4) For a given voxel (node), nodal FCS was computed as the sum of weights of its connections with other voxels. We conservatively restricted our analysis to positive correlations above the threshold of *r* = 0.2. Furthermore, to calculate the ALFF measure at each voxel within the GM mask, the time series of each voxel was transformed to the frequency domain through fast Fourier transform analysis. The power spectrum was then computed. The mean value of the square root of activity in the low-frequency band (0.01–0.08 Hz) was computed as the ALFF. Finally, individual voxel-wise FCS and ALFF maps were standardized by dividing the full-brain mean values and further spatially smoothed (FWHM = 6 mm), respectively.

### Tracking Spatial Organization of Dynamic Functional Network Across Time Windows

For dynamic functional maps (FCS or ALFF), we tracked the FCS/ALFF-sDFN across time windows (Figure 1B) through group spatial ICA analysis (GIFT software: http://mialab.mrn.org/software/gift/). The low dimension was used to define the stable sDFN components. Twenty independent components (from the 1000 Functional Connectomes Project) (33) were initially extracted from all functional maps using the Infomax algorithm, which was repeated 20 times in ICASSO (http://research.ics.tkk.fi./ica/icasso). Nine sDFNs were firstly selected on the basis of the largest spatial correlation (SC) analysis with the corresponding template, including sensorimotor network (SMN), visual network (VN), auditory network (AN), DMN, salience network (SN), basal ganglia network (BGN), left frontoparietal network (LFPN), right frontoparietal network (RFPN), and dorsal attention network (DAN). The detailed steps were shown as the following: (1) One sample *t*-test was performed for each component. (2) The mask was calculated through family-wise error (FWE) correction. (3) For each template of RSN, Spearman correlation was performed between template matrix (*1 by N* matrix, *N*: the number of voxels within the whole brain) and 20-masked-component matrix (*1 by N* matrix, *N*: the number of voxels within the whole brain) respectively. (4) For each RSN template, the related sDFN was selected based on the largest SC score. Secondly, the selected nine components were visually inspected. Then, individual dynamic time courses and spatial maps of sDFN were reconstructed for each subject using the spatial-temporal regression approach. The dynamic time course of sDFN represents the variability of the functional network across the various time windows for each subject. The spatial map of sDFN represents the contribution of each voxel to the variability of the functional network across time windows.

A two-sample *t*-test was performed in each spatial map of sDFN between schizophrenic and healthy subjects [*p* < 0.05, cluster-level false discovery rate (FDR) corrected] with gender, GM value, age, and FD as covariates. In addition, the connectivity was computed between the dynamic time courses of two sDFNs by using Pearson correlations for each subject (Figure 1C). The Fisher *r*-to-*z* transformation of these correlations was performed. Two-sample *t*-tests were also performed for all potential connections among sDFNs with gender, GM value, age, and FD as covariates, with a statistical significance level of *p* < 0.05 corrected by FDR.

### Correlations With Pathological Factors

We examined the association between the PANSS scores and changed dFC within each sDFN and connectivity between sDFNs, respectively, with gender, GM value, age, and FD as covariates in schizophrenic subjects. FDR correction (*p* < 0.05) was used for multiple comparisons.

### Reproducibility of sDFN Across Window Sizes Through Intra-class Correlation

To assess the reproducibility of the spatial pattern of sDFN and the connectivity between sDFNs across a range of window sizes, sliding window-based FCS and ALFF calculations were performed in all subjects with a range of window sizes, respectively, including 100, 110, 120, 130, and 140 s. The score of SC was assessed between FCS/ALFF-sDFN and the corresponding network template. The connectivity between the time series of sDFNs was also calculated. Then, the ICC was computed for scores of SC and connectivity between sDFNs to quantify the reproducibility, respectively (34):

$$\begin{array}{l} ICC=\frac{\sigma_{b}^{2}}{{\sigma_{b}^{2}+\sigma }_{w}^{2}} \end{array}$$

where $\sigma_{b}^{2}$ represents between-subject variability and $\sigma_{w}^{2}$ is the variability within subjects. An ICC value close to 0 indicates poor reproducibility, and a value close to 1 represents excellent reproducibility. Moreover, we compute statistical significance using the *F*-statistic (35):

$$\begin{array}{l} F=\frac{1+ICC\left(k-1\right)}{1-ICC} \end{array}$$

where *k* is the number of repeated windows. The corresponding *p*-value of the *F*-statistic was then computed for degrees of freedom *df* 1 = 216 and *df* 2 = 868. Then, FDR correction (*p* < 0.05) was used for multiple comparisons for SC and connectivity between sDFNs, respectively.

## Results

### Demographics and Patient Clinical Characteristics

Six schizophrenic subjects and four healthy controls with head motion scans exceeding 3 mm or 3° rotation were excluded. Additionally, three healthy controls were excluded because of deficiency of data by visual evaluation. Finally, 96 schizophrenic subjects and 121 healthy controls were included in this study. Schizophrenic subjects' clinical characteristics, including illness duration, medication dosage, and PANSS scores, are presented in Table 1. Both groups did not differ significantly in gender, age, and head motion (FD). The normalized GM of schizophrenic subjects was decreased compared to healthy controls (Table 1).

**Table 1:** Demographic and clinical characteristics of the participants.

	**Patients with schizophrenia**	**Healthy controls**	***p***
Gender (male/female)	68/28	80/41	0.46^a^
Age (years)	41.72 ± 11.91	39.92 ± 13.99	0.31^b^
Head motion (FD)	0.056 ± 0.05	0.047 ± 0.03	0.07^b^
Normalized GM	0.43 ± 0.03	0.45 ± 0.02	0.001^b^
Disease duration (years)	15.68 ± 10.89	-	-
Chlorpromazine equivalents (mg/day)^c^	336.54 ± 163.58		
PANSS-positive score^d^	13.42 ± 5.90	-	-
PANSS-negative score^d^	20.67 ± 6.02	-	-
PANSS-global score^d^	28.20 ± 5.82	-	-
PANSS-total score^d^	62.30 ± 13.18	-	^−^

*Indicated values are shown mean ± standard deviation*.

*PANSS, Positive and Negative Symptom Scale; GM, gray matter*.

a *Indicates the p-values from the comparison analysis (chi-square-test)*.

b *Indicates the p-values from the comparison analysis (two-sample t-test)*.

c *Data of 70 schizophrenic patients available*.

d *Data of 64 schizophrenic patients available*.

### Spatial Organization of the Dynamic Functional Network in FCS/ALFF

**Figure 2:**
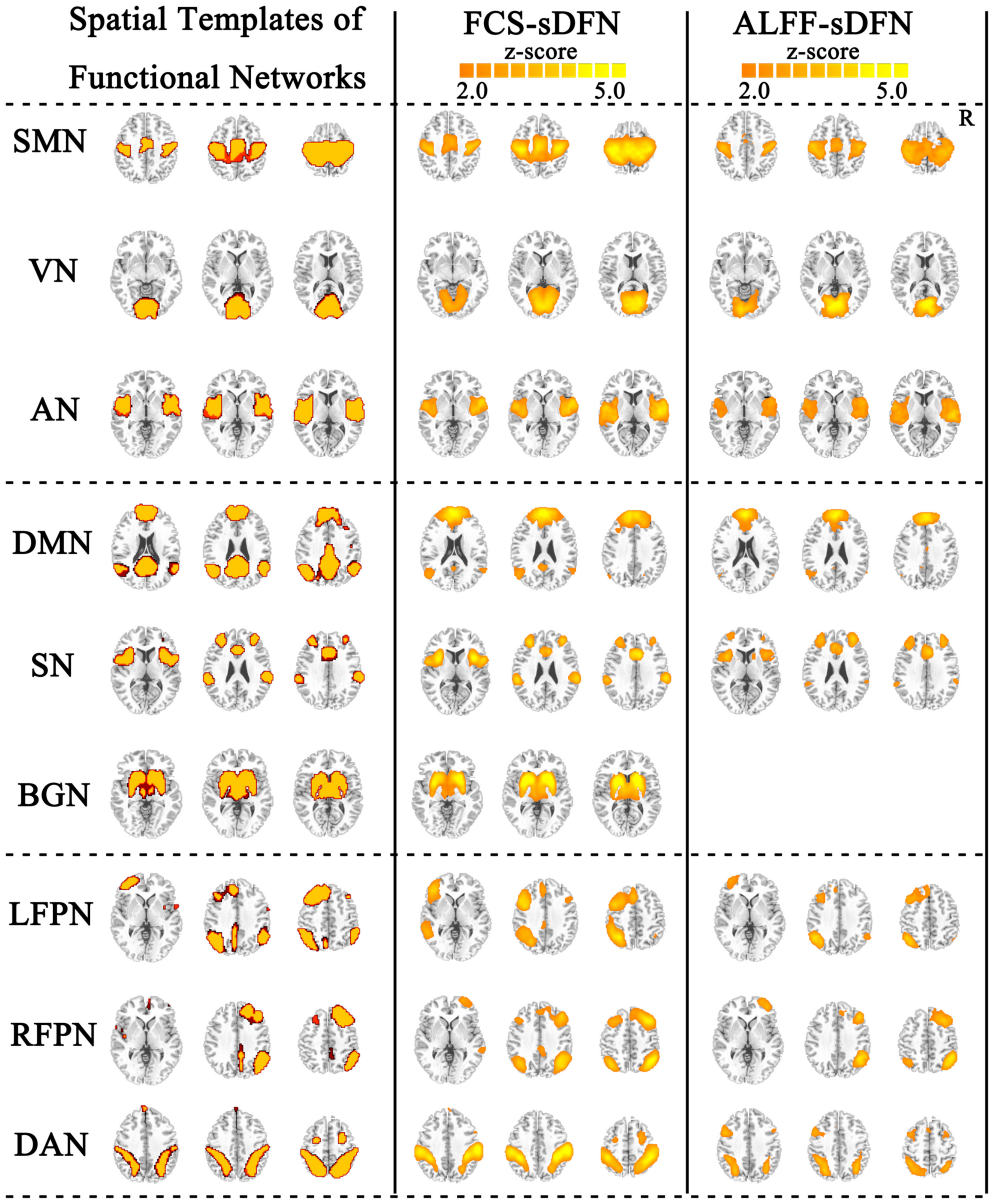
Nine identified intrinsic spatial organization of dynamic functional networks (sDFNs). The first column includes the spatial template of nine resting-state functional networks (RSNs). The second and third columns denote the related FCS-sDFN and ALFF-sDFN, respectively. FCS, functional connectivity strength; ALFF, amplitude of low-frequency fluctuations.

FCS/ALFF-sDFNs showed widespread functional architecture in both groups. As shown in Figure 2, the temporal variability of whole-brain voxels across the various time windows was spatially heterogeneous. We found that the FCS-sDFN showed nine specific patterns of network organization in the human brain, namely, SMN, VN, AN, DMN, SN, BGN, LFPN, RFPN, and DAN. ALFF-sDFN also showed eight specific patterns of network organization, namely, SMN, VN, AN, DMN, SN, LFPN, RFPN, and DAN. The BGN was not observed in ALFF-sDFN. Importantly, the spatial pattern of each sDFN was similar with the related RSN; the largest SC score is shown in Table 2.

**Table 2:** Largest spatial correlation score (SCS) between nine template of resting-sate networks and FCS-sDFN, as well as ALFF-sDFN.

**Template of resting-state network**	**SCS of FCS-sDFN (*r* score)**	**SCS of ALFF-sDFN (*r* score)**
Sensorimotor network	0.603	0.479
Visual network	0.438	0.388
Auditory network	0.422	0.371
Default model network	0.320	0.219
Salience network	0.502	0.449
Basal ganglia network	0.485	–
Left frontoparietal network	0.334	0.271
Right frontoparietal network	0.318	0.242
Dorsal attention network	0.409	0.351

**Figure 3:**
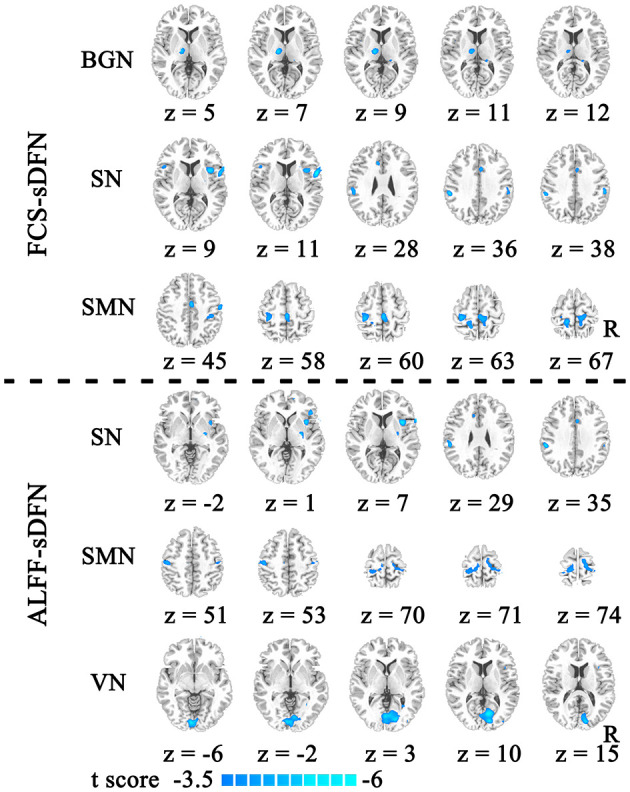
The reduced dynamic functional connectivity (dFC) within the spatial organization of dynamic functional network (sDFN) in schizophrenia. Decreased dFCs within FCS-sDFN were displayed above the dotted line, including the basal ganglia network (BGN), salience network (SN), and sensorimotor network (SMN). Under the dotted line, reduced dynamic functional activity within ALFF-sDFN is shown, including SN, SMN, and visual network (VN). FCS, functional connectivity strength; ALFF, amplitude of low-frequency fluctuations.

The schizophrenic subjects showed decreased dynamic FCS/ALFF in sDFN compared to healthy subjects. Within FCS-sDFN, abnormal dynamic FCS was observed in BGN (thalamus), SN (anterior insula, anterior cingulate cortex, and supramarginal region), and SMN (post-central and pre-central gyri) in schizophrenic subjects. A similar reduced dynamic ALFF was also observed in ALFF-sDFN in patients with schizophrenia, including SN (anterior insula, and supramarginal region), SMN (post-central and pre-central gyri), and VN (calcarine and lingual regions) (Table 3 and Figure 3).

**Table 3:** Significant reduced dynamic FCS/ALFF in sDFN of schizophrenic subjects.

		**MNI coordinates**				
**Component**	**Regions**	***x***	***y***	***z***	***T*-score**	**Cluster voxels**
BGN-FCS	Left thalamus	−12	−18	6	−5.269	79
SN-FCS	Right insula	37	10	11	−5.782	83
	Right Rolandic operculum	57	3	12	−5.671	163
	Anterior cingulate cortex	−9	21	28	−5.477	57
SMN-FCS	Left post-central	−18	−38	67	−5.267	132
	Right post-central	3	−30	58	−4.935	128
SN-ALFF	Right insula	39	7	7	−5.686	87
SMN-ALFF	Right pre-central	15	−27	74	−5.483	119
	Left post-central	−15	−33	78	−4.877	97
VN-ALFF	Right calcarine	9	−72	6	−5.713	390

*FCS, functional connectivity strength; ALFF, amplitude of low-frequency fluctuations; BGN, basal ganglia network; SN, salience network; SMN, sensorimotor network; VN, visual network*.

**Figure 4:**
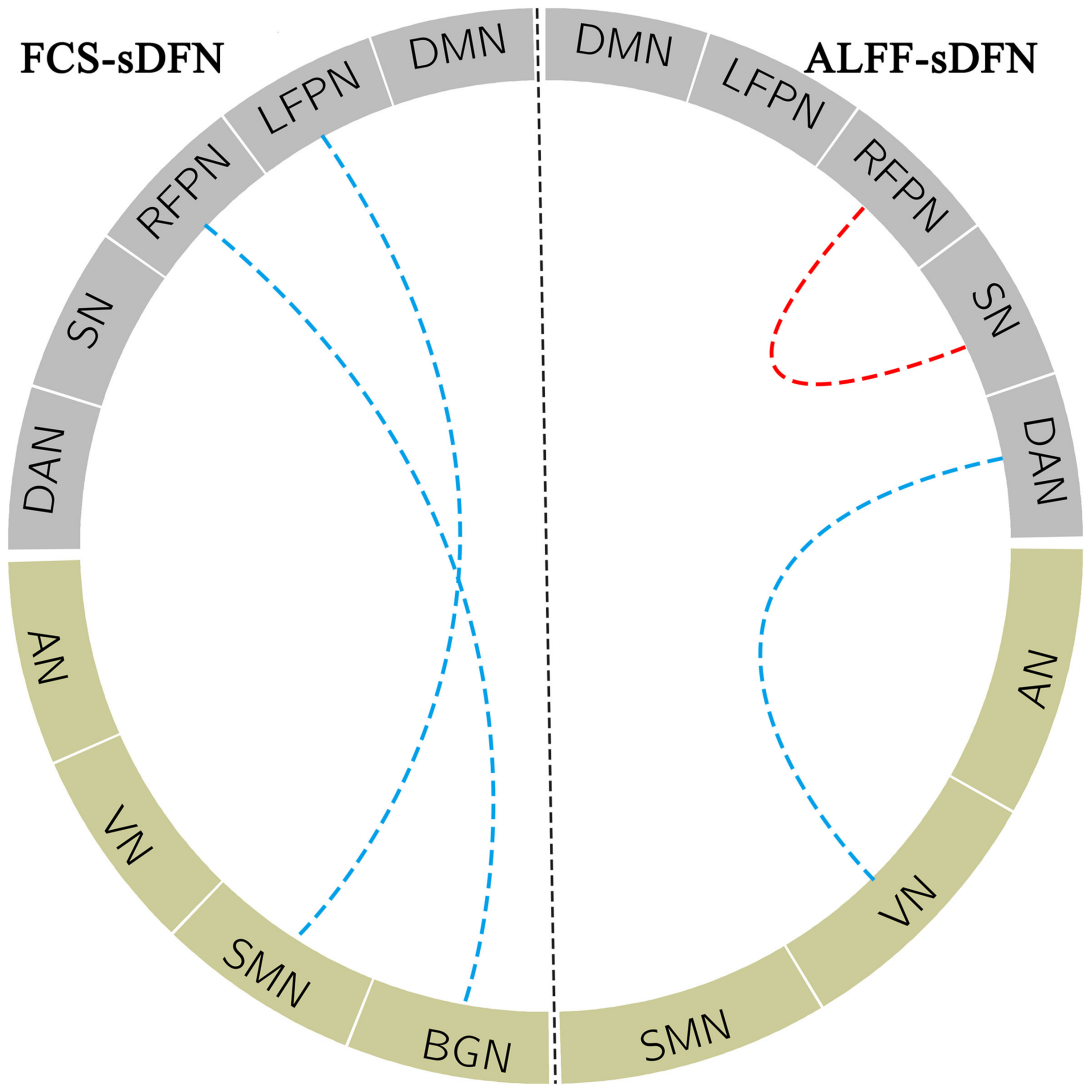
The blue dotted line within the FCS_sDFN section denotes decreased negative connectivity between sDFNs in schizophrenic patients compared to healthy controls. Within ALFF_sDFN, the red dotted line denotes reduced positive connectivity; the blue dotted line represents the decreased negative connectivity between sDFN in schizophrenic subjects compared to healthy controls. FCS, functional connectivity strength; ALFF, amplitude of low-frequency fluctuations; sDFN, spatial organization of dynamic functional network; DMN, default model network; LFPN, left frontoparietal network; RFPN, right frontoparietal network; SN, salience network; DAN, dorsal attention network; AN, auditory network; VN, visual network; SMN, sensorimotor network; BGN, basal ganglia network.

A significant connectivity between FCS/ALFF-sDFN, including positive and negative correlations, was observed in subjects. Depending on the connectivity pattern, these sDFNs could be divided into two clusters: positive connectivity within clusters and negative connectivity between clusters. One cluster comprised DMN, FPN, SN, and DAN; the other cluster comprised AN, SMN, VN, and BGN. In FCS-sDFN, a reduced negative connectivity was observed between LFPN and SMN and between BGN and RFPN in schizophrenic subjects (Figure 4). An altered connectivity was also found in ALFF-sDFNs in schizophrenia (Figure 4), such as decreased positive connectivity (between RFPN and SN) and a reduction of negative connectivity (between DAN and VN). Similar results were also observed in the other two window lengths (i.e., 120 and 140 s).

### Correlations With Pathological Factors

**Figure 5:**
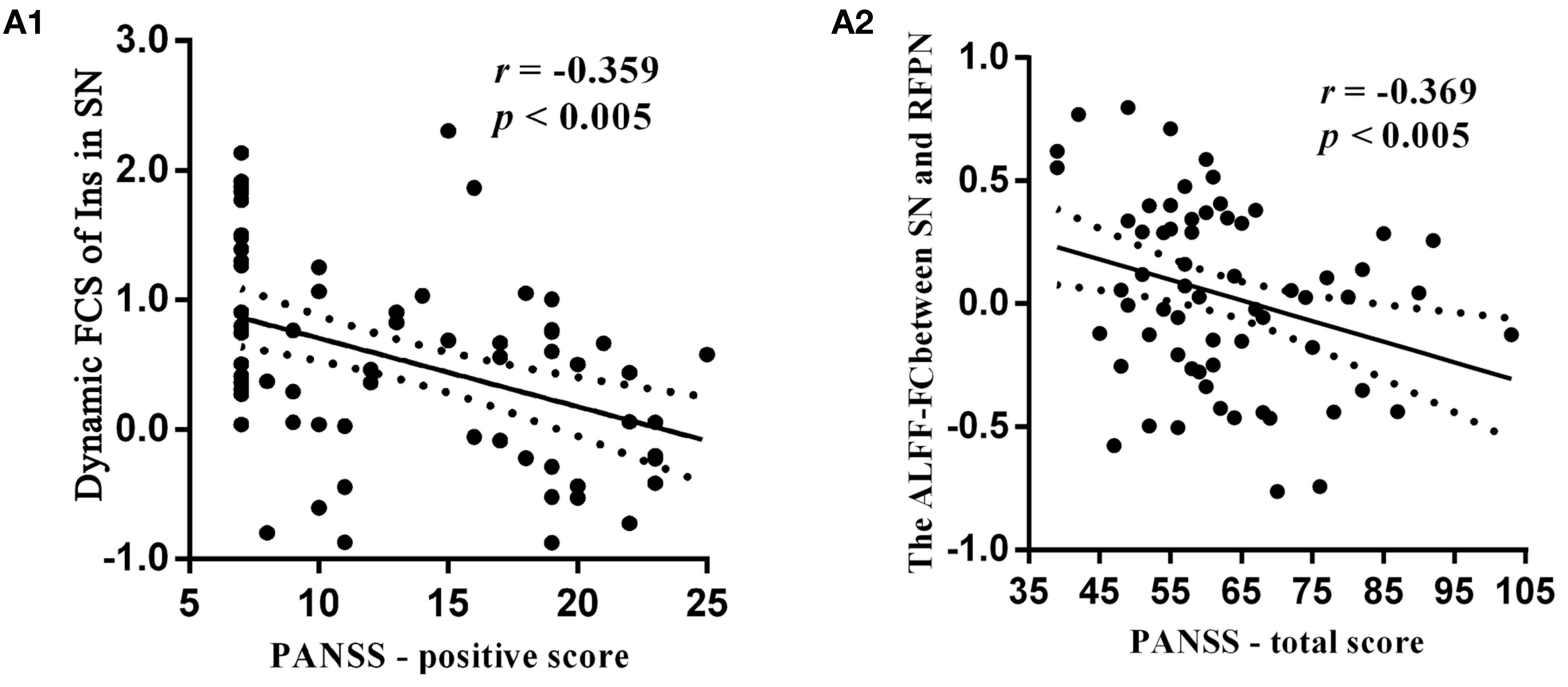
Relationship between clinical symptoms of schizophrenic subjects and dynamic functional connectivity (dFC) within the spatial organization of dynamic functional network (sDFN), and connectivity between sDFNs respectively. **(A1)** Denotes a negative relationship between the dynamic FCS value of right insula (Ins) within SN-sDFN and p-PANSS scores. **(A2)** Denotes the negative relationship between total score of PANSS and connectivity between ALFF-sDFN (SN and RFPN). FCS, functional connectivity strength; ALFF, amplitude of low-frequency fluctuations; PANSS, Positive and Negative Symptom Scale.

The relationship between clinical score and sDFN could further support the contribution of aberrant sDFN to the pathophysiology of schizophrenia. We observed a negative correlation between PANSS scores and dFC within the FCS-sDFN of schizophrenic subjects: PANSS-P score and insula within SN (*r* = −0.359, *p* < 0.005, Figure 5A1). We also found a negative correlation between the PANSS-total score and connectivity between ALFF-sDFNs (SN-RFPN, *r* = −0.369, *p* < 0.005, Figure 5A2) in schizophrenic subjects. No significant relationship was observed between PANSS scores and other altered dFCs. No significant relationship was observed between altered dFC and GM value, as well as FD score.

### Reproducibility of sDFN Through ICC

**Figure 6:**
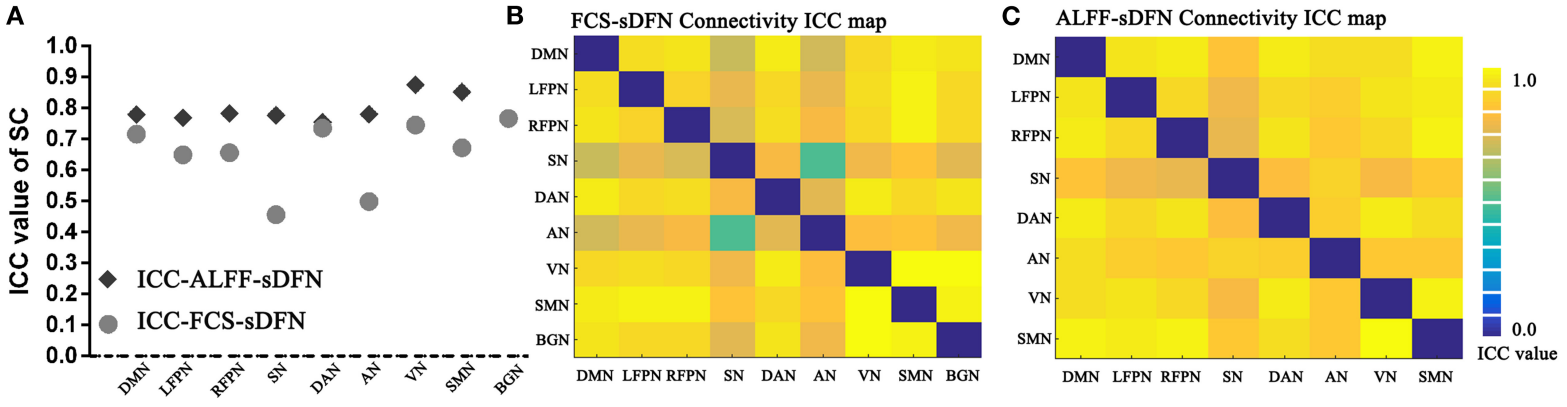
Intra-class correlation (ICC) maps of spatial correlation (SC) and connectivity between sDFN across a range of window sizes in subjects. **(A)** Shows the ICC values of SC, including SC of FCS-sDFN and SC of ALFF-sDFN. **(B)** Denotes the ICC values of connectivity between FCS-sDFN. **(C)** Denotes the ICC values of connectivity between ALFF-sDFN.

Both SC and connectivity between sDFNs have significant high reproducibility across a range of window sizes in subjects (Figure 6A: SC, Figure 6B: connectivity). The SC-ICC value of FCS-sDFN ranges from 0.45 to 0.76 (mean value: 0.66, standard deviation: 0.10). The connectivity–ICC value of FCS-sDFN ranges from 0.47 to 0.88 (mean value: 0.77, standard deviation: 0.08). The ALFF-sDFN ICC values of SC and connectivity between sDFN are higher than the ICC score of FCS-sDFN: SC-ICC: 0.75–0.87, mean ± standard deviation: 0.79 ± 0.04; connectivity–ICC: 0.93–0.96, mean ± standard deviation: 0.95 ± 0.007.

## Discussion

Consistent with our hypothesis, we found that voxels dynamically switched their functional network across different time windows, named as sDFN. The BGN was not identified by ALFF-sDFN. BGN mainly allows for fast local events. The dynamic power of different regions within BGN might be associated with its fast dynamic local activity. Our findings might indicate that the stable BGN-sDFN could be easier identified by its local whole-brain dFC than its local dynamic power. Furthermore, the schizophrenic subjects displayed decreased dynamic FCS/ALFF in multi-perception sDFN as well as a deficient connectivity between sensory and high-order FPNs. Critically, these aggravated dFCs were related with higher severity of pathological positive and total symptoms of schizophrenic patients. Resonating with recent theories and studies (20, 36, 37), these findings highlight the disrupted dynamic sensory high-order connectivity in schizophrenia and further provide the critical role of altered dynamic functional integration of higher-order processes, helping to understand the pathophysiology of schizophrenia.

### sDFNs Form the Functional Foundation of RSNs

In the human brain, the functional connectors (e.g., fronto-parietal regions) may switch between different functional systems for higher cognitive flexibility during tasks (38, 39). Resting-state time-dependent functional networks may also reflect the spontaneous transitions among the potential functional coordinating configurations, which would provide a fast response to extrinsic stimuli (40). Empirical and modeling studies have been suggested that changes in static functional connectivity can be traced to changing patterns of communication dynamics (41). Thus, in the current study, we defined the sDFN through combining sliding window-based dFC and group ICA approaches to assess the spatial architecture of dFC across the various time windows in the human brain. We observed that the RSNs dynamically switched their functional networks across time, suggesting a time-dependent modular structure of the dFC in the human brain. The spatial maps of sDFN from ICA imply the contribution of each voxel to its sDFN. Specifically, these findings remained almost unchanged when using reproducibility measurements across lengths of window. The correction and interpretation of head motion should also be carefully handled in future studies because it may reflect individual variability in functional organization (42). These results suggest that the remarkable intra-subject sDFN observed here was not dominantly driven by the window length. Therefore, our novel findings highlighted that the sDFN during rest might form the intrinsic functional foundation for individual flexible task function or extrinsic stimulus.

### Reduced dFC in Sensory and Perception sDFN

Schizophrenic subjects showed decreased dFC within sensory and perceptual sDFN, including FCS (i.e., SMN) and ALFF (i.e., SMN and VN). Deficits of sensorimotor processing and multisensory functional connectivity integration, first investigated by Kraepelin and Bleuler, were well-documented as possible pathophysiological mechanisms in schizophrenic subjects (43, 44). Heightened dFC was then found within sensory and perceptual networks, such as VN (lingual gyrus and lateral occipital cortex), BGN, and SMN (pre-central and post-central gyri) in schizophrenia patients, which indicate that these perception regions are over local functional interacting across time (20). Furthermore, the increased time-varying connectivity of sensory and perceptual regions may result in spreading of disrupted internal and external sensory information to the distant high-order regions in schizophrenia (37, 45). In this study, we found decreased dFC in sensory networks (i.e., SMN, VN, and BGN) across the various time windows in schizophrenia patients. Combining our novel findings and previous studies, these decreased sensory sDFN may lead to reduced primary perceptual functional foundation and also contribute to the deficits of sensory high-order functional connectivity in schizophrenia.

SN was regarded as a whole unit to monitor internal and external salient information in the human brain (46). SN is one critical functional network that provides the basis of the representations of interoceptive awareness and external environment (46). Many symptoms (e.g., deficient facial affect processing and auditory affect processing) observed are involved with these aberrant static functions in schizophrenic subjects (47). Enhanced variability of functional connectivity within SN (e.g., insula and anterior cingulate cortex) in schizophrenia also supports the inappropriate function of sequential integration of interoceptive awareness with external perceptual stimulus in schizophrenic subjects (48, 49). This may eventually contribute to positive symptoms (e.g., hallucinations) (47). The deficient dFC of SN may reduce the functional foundation for regulating salient information and maintaining the integrated self in schizophrenic subjects.

In the present study, the deficient sDFN within primary perceptual regions and monitoring networks provided novel evidence to support altered dFC in processing external or internal sensory information in schizophrenia. These decreased dFCs may form deficient intrinsic function that may affect the intrinsic brain state, contributing to positive symptoms in schizophrenic subjects. Furthermore, recent studies hypothesized that abnormal sensory processing underlay cognitive impairment, in turn affected by high-order cognitive dysfunction (7, 8, 20). Unfortunately, the aberrant dFC between sensory and cognitive processes is still not regarded as the intervention target.

### Involvement of the Difference of High-Order Dynamic Function and Pathology

Neural processes depend on dynamic functional interactions between regions or networks in the human brain, which is thought to be instrumental for integrating and processing information in the course of behavior and cognition (41, 50, 51). These dFCs are coordinated through top-down projections from high-order functional networks, mostly located in FPN (52). Specifically, FPN could modulate sensory and other association networks to manage internal and external information (39, 53, 54). Combining with abnormal dFC within perceptual regions, altered static and dFCs of FPN revealed that dysfunctional modulation of FPN on internal states and salient stimuli may result in confusion about self-related and unrelated information in schizophrenic subjects (4, 9, 55, 56). In the present study, we did not find aberrant dFC within FPN in schizophrenic subjects, whereas decreased distant connectivity was observed in patients between FPN and distant networks, including SN, BGN, and SMN. Our findings may reveal that dysfunctional dFC between high-order and perceptual and monitoring systems may lead to less flexible function to modulate the internal and salient stimuli of schizophrenia.

In recent years, it has been hypothesized that the bottom-up sensory and perceptual dysfunctions may be a bottleneck in higher-level cognitive processing (7) and have been attributed as the causal role in clinical symptoms of schizophrenia (57, 58). Using the present advanced approach of sDFN, reduced dFC was consistently observed within primary sensory networks and SN, and between sensory and FPN in schizophrenic subjects. Critically, decreased dFCs of SN were tightly linked with severity of positive symptoms of schizophrenic subjects. The total PANSS score was also associated with decreased connectivity between SN and RFPN in schizophrenic subjects. Together, these findings verified the hypothesis that schizophrenia is more due to perceptual incoherence (2, 3, 7), which might be the causal role in clinical symptoms of schizophrenic subjects.

## Limitations

While our novel findings highlighted that the sDFN during rest might form the intrinsic functional foundation for individual flexible task function or extrinsic stimulus compared to previous dynamic functional studies, the main limitations of this study should be acknowledged. First, the main limitation is the effect of antipsychotic drugs. While the altered dFC was not associated with medication in this study, we cannot eliminate completely the potential confounding effects of medication on schizophrenia. Second, due to the cross-sectional research design of this study, we cannot establish the developmental trajectories of altered sDFN in schizophrenia. This work should be designed in the future study. Third, to assess the stable sDFN, low dimension of 20 independent components was determined in the group spatial ICA analysis. The higher-order ICA model might give more detailed information related to the neuropathology of schizophrenia. Fourth, our findings verified the hypothesis that schizophrenia is more due to perceptual incoherence. The validation analysis should be done in future research through the rTMS on SMN.

## Conclusion

Through sDFN analysis, we found that the spatial architectures of sDFN and RSNs were largely overlapped in the human brain. This indicates that the sDFN during rest may form the intrinsic functional foundation for dynamically endogenous or exogenous perturbations. In schizophrenic subjects, both decreased dFC in perceptual sDFNs and reduced connectivity between sensory and FPN were observed. These findings support the hypothesis that aberrant perceptual and high-order functional network is related with the pathophysiology of schizophrenia. Moreover, our results may also partly reflect the abnormal intrinsic ongoing fluctuations of functional connectivity during perception processing and modulation between perceptual and FPN in schizophrenia, letting us understand the underlying neuropathology of schizophrenia from a new perspective.

## Data Availability Statement

The raw data supporting the conclusions of this article will be made available by the authors, without undue reservation.

## Ethics Statement

The studies involving human participants were reviewed and approved by Clinical Hospital of Chengdu Brain Science Institute. The patients/participants provided their written informed consent to participate in this study.

## Author Contributions

HH, CL, MD, and DY made a substantial contribution to the conception, design of the experiment, drafting and revision of the article, and gave the final approval of the version to be published. CH, HH, BB, GY, and MH made a substantial contribution to the analysis and interpretation of the data, revision of the article critically, and gave the final approval of the version to be published. GY, BB, and JD made a substantial contribution to the acquisition, interpretation of the data, and gave the final approval of the version to be published. All authors contributed to the article and approved the submitted version.

## Conflict of Interest

The authors declare that the research was conducted in the absence of any commercial or financial relationships that could be construed as a potential conflict of interest.

## Publisher's Note

All claims expressed in this article are solely those of the authors and do not necessarily represent those of their affiliated organizations, or those of the publisher, the editors and the reviewers. Any product that may be evaluated in this article, or claim that may be made by its manufacturer, is not guaranteed or endorsed by the publisher.
